# Self-concept in fairness and rule establishment during a competitive game: a computational approach

**DOI:** 10.3389/fpsyg.2015.01321

**Published:** 2015-09-08

**Authors:** Sang Ho Lee, Sung-Phil Kim, Yang Seok Cho

**Affiliations:** ^1^Department of Psychology, Korea UniversitySeoul, South Korea; ^2^Department of Human and Systems Engineering, Ulsan National Institute of Science and TechnologyUlsan, South Korea

**Keywords:** fairness, altruism, reciprocity, self-concept, computational model, economic game

## Abstract

People consider fairness as well as their own interest when making decisions in economic games. The present study proposes a model that encompasses the self-concept determined by one's own kindness as a factor of fairness. To observe behavioral patterns that reflect self-concept and fairness, a chicken game experiment was conducted. Behavioral data demonstrates four distinct patterns; “switching,” “mutual rush,” “mutual avoidance,” and “unfair” patterns. Model estimation of chicken game data shows that a model with self-concept predicts those behaviors better than previous models of fairness, suggesting that self-concept indeed affects human behavior in competitive economic games. Moreover, a non-stationary parameter analysis revealed the process of reaching consensus between the players in a game. When the models were fitted to a continuous time window, the parameters of the players in a pair with “switching” and “mutual avoidance” patterns became similar as the game proceeded, suggesting that the players gradually formed a shared rule during the game. In contrast, the difference of parameters between the players in the “unfair” and “mutual rush” patterns did not become stable. The outcomes of the present study showed that people are likely to change their strategy until they reach a mutually beneficial status.

## Introduction

People have motivation to care about other people (Hume, 1740; Smith, 1759; MacIntyre, 1967), unlike the assumption of traditional economic models that people are exclusively motivated to pursue their own material interest (Kahneman et al., 1986). One critical factor affecting such other-regarding interest is “fairness.” People are willing to sacrifice their own interest if it is considered fair (Rabin, 1993). Some studies suggest “altruism” as a source of fairness (Eckel and Grossman, 1996), while other studies propose “reciprocity” (Cox et al., 2007). Altruism stands for the behavior of people who perceive an equal distribution of welfare among people as fair. Human behavior in laboratory experiments of public goods games evidences altruism as a factor in decision-making (Andreoni, 1988, 1995). In those experiments, people did not necessarily contribute to public goods at all to maximize their own interest, but most people chose to sacrifice their payoff to some degree to increase the total public payoff (Dawes and Thaler, 1988). In addition to altruism, reciprocity has long been studied as a factor of fairness. People often sacrifice their own welfare to help those who are being kind (Marwell and Ames, 1981; Güth et al., 1982; Van de Kragt et al., 1983; Isaac et al., 1984; Kim and Walker, 1984; Andreoni, 1988; Isaac and Walker, 1988; Orbell et al., 1988) or to punish others those who are being harmful (Goranson and Berkowitz, 1966; Greenberg, 1978; Güth et al., 1982; Kahneman et al., 1986; Roth et al., 1991; Thaler, 1999). For example, in an ultimatum game, people often reject to receive a smaller payoff than their counterparts and rather choose an allocation in which neither player receives any payoff (Slonim and Roth, 1998).

This long line of evidence supports that fairness is a critical factor of decision-making (Cox and Deck, 2005; Cox et al., 2008). To consolidate our understanding of fairness in human behavior, various computational models have been developed (Rabin, 1993; Levine, 1998; Fehr and Schmidt, 1999; Charness and Rabin, 2002; Dufwenberg and Kirchsteiger, 2004; Cox and Sadiraj, 2005; Cox et al., 2007). Fehr and Schmidt (1999) proposed a model featuring inequality averseness of individuals to explain the altruistic behaviors that deviate from pure self-interest. According to their model, people prefer a “fair” distribution of income so that little differences between their own income and other people's income exist. The Fehr–Schmidt model and its variations are consistent with many experimental results showing the other-regarding interest of people (e.g., Bolton and Ockenfels, 2000). However, this simple preference model does not capture the intentions of people behind their behaviors in different contexts. For example, in a mini-ultimatum game, when the first mover chooses between two alternatives, identical offers are evaluated differently by the second mover according to possible alternatives (Falk et al., 2008). People are willing to accept an unequally distributed payoff offer when the alternative is even more unfair. That is, if a seemingly unfair action is conducted with good intention, people perceive the action as less harmful.

Capturing the intention of other people is especially important in the models based on reciprocity. A model developed by Rabin (1993) considers the intentions of other people to find fairness equilibria. In his model, the kindness of people is determined by their intention. Even a harmful action of a person does not induce negative reciprocity of another to punish him/her if there is no better alternative. In Rabin's fairness equilibria, such reciprocal motivation is emphasized rather than the equal distribution of income. Many recent studies support the effects of reciprocity on people's preference rather than simple inequality averseness (e.g., Blount, 1995; Brandts and Charness, 2000). However, higher-order beliefs about other people's intentions assumed in the model are too complex to define in behavioral data, because they are not reported directly in most experiments and also because there are many constraints in reporting the feeling at the moment (Robinson and Clore, 2002). To avoid the complications of the equilibrium model involving higher-order beliefs, Cox et al. (2007) introduced a model of reciprocity and fairness that simplifies contexts affecting people's preference. Their model introduces “emotional states” that reflect reciprocity without the assumption of complex beliefs. A kind behavior induces positive emotional state while a harmful behavior leads to negative emotional state. People with a positive emotional state are inclined to give others a positive payoff and those with a negative emotional state prefer to give a negative payoff to others. Cox et al.'s (2007) model is easily applicable to various economic games because of its tractability, compared to models that assume higher-order beliefs.

Most economic games have a bilateral flow of benefit in which the players' payoff is determined by their joint action, not by an action of a single player. That is, the structure of reciprocity is bilateral (Molm, 2010), and therefore both players can benefit or harm each other. Thus, the self-concept of a player has to be considered in addition to the reciprocity that reflects the kindness of another player. People consider the morality or kindness of their own behavior to define their self-concept (Dunning, 2007). People's self-worth is lowered after they harm another person and they become more altruistic to compensate the immoral behavior (Carlsmith and Gross, 1969). Similarly, people with increased self-worth after a kind behavior are more likely to engage in an immoral behavior (Monin and Miller, 2001; Khan and Dhar, 2007). These phenomena are attributed to the monitoring and balancing of self-concept that maintains one's self-worth to an ideal level in which an individual feels the most comfortable. If people feel that their self-worth is lower than their standard, they try to increase their self-worth by a positive behavior. In the same way, they lower their self-worth with a negative behavior when their self-worth is higher than their standard (Sachdeva et al., 2009).

Because the previous models regarding reciprocity do not capture the self-concept of an individual, the present study proposes a model to incorporate self-concept into the model of fairness. In the previous models, reciprocity emerges only from the concept about other people. However, our model determines reciprocity by a comparison between the self-concept and the concept about others. If an individual has relatively negative self-worth compared to his/her concept about the other, he/she has positive reciprocity and is inclined to help the counterpart. That is, people in the present model choose to help their unkind counterparts if they perceive themselves more unkind than the counterparts. In addition, the present model assumes that the sense of reciprocity is more volatile than the calculation of relative payoffs so it better captures the dynamic changes of reciprocity during a social interaction. People may easily forget past negative reciprocity in the face of kindness of the former counterpart, while the difference between material payoffs is constant (Komorita et al., 1991).

In the present study, we compared the proposed model with the previous models regarding the fairness and reciprocity and showed whether the model based on the sense of reciprocity and the self-concept explains the dynamic behaviors in an iterated competitive game comparable to the models based on the inequality averseness of material payoff. If one's own kindness affects one's next behavior, the proposed model that focuses on the self-concept will predict people's behavior better than the other models of fairness. In addition, we aimed to investigate the process of rule establishment through changes in the parameters of the model. If the players share a same structure of a utility function, as various models suggested (e.g., Weitzman, 1965), establishing a rule based on the shared utility function would be beneficial to the players because strategies that maximize their utility are similar among them. For example, if all players share the utility function defined by inequality averseness, establishing a rule that leads to an equal distribution of income maximizes the utility of every player. A rule is reflected by the parameters of a model that adjusts behavior according to the parameter values.

People often change their preferences after a social interaction such as discussion or negotiation (Kaplan and Miller, 1987). Changing a personal decision rule is often a result of consensus between people. Once a consensus is achieved, they share a common rule that governs the joint behavior (Penrod and Hastie, 1980; Stasser and Davis, 1981; Hastie et al., 1983). It is hypothesized that the players would show undefined behavioral patterns at first, but players with a similar utility function would gradually reach consensus and play based on the rules. This hypothesis was tested by non-stationary parameter analysis that calculates model parameters in continuous time windows, instead of fitting the parameters once with the whole data in an experiment. The changes of the parameters that lead to certain behaviors are shown by this method. If the players gradually establish a rule between them, the fitted parameters of each player would reflect the rule as the game continues. For example, the “initial benevolence” parameter in Cox et al.'s (2007) model induces kind behavior when the parameter value is high. Thus, the parameter values of the players who reach a consensus to behave kindly to each other will increase during a game.

**Figure 1:**
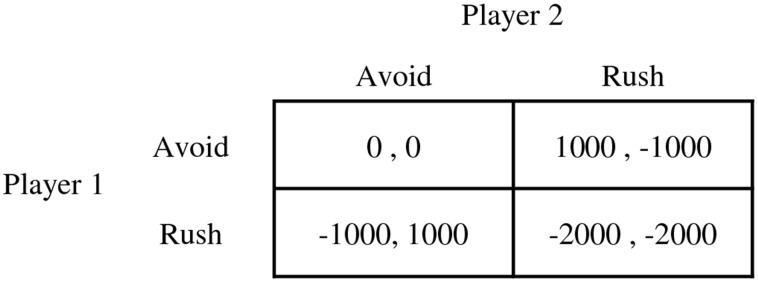
**Reward structure of the chicken game in the present study (in KRW)**.

An iterated chicken game was used in the present study to obtain behavioral data that reflect self-concept as well as reciprocity (e.g., Jankowski, 1990). In a chicken game, each of the two players controls a car that rushes toward each other. A player who avoids becomes a “chicken” and loses certain amount of reward to the opponent. If nobody avoids and the cars crash, they both lose great amount of reward. Nobody loses or gains anything when both of the players choose to avoid. The reward structure of the game used in the present experiment is shown in Figure 1. The chicken game is competitive in its nature and has no single fairness equilibrium or Nash equilibrium. One needs to rush to have a chance to gain more benefit than the opponent, but it has a high risk of crash. When both players keep avoiding, they always have an incentive to betray and rush to earn more. Therefore, this game allows us to observe dynamic behavioral patterns that do not easily allow consensus between players. Thus, it is appropriate to see the gradual process of rule establishment between the players, while varied patterns are expected through the process. Furthermore, it is free from the beliefs about intentions because the rush is always harmful to the other player and the avoidance is always helpful; unintended harm, or kindness does not exist in the game. It makes the application and comparison of various models quite easy and simple.

## Material and methods

### Experimental procedures

#### Participants

Seventy two undergraduate students (mean age = 24.07, 29 females) at Korea University participated in the study for the monetary reward of KRW 10,000 (about 9 US dollars). All participants had normal or corrected to normal vision and reported no neurological or psychiatric problems. The present experiment was approved by the Institutional Review Board at Korea University (KU-IRB-13-66-A-1) and all participants gave written informed consent in accordance with the Declaration of Helsinki.

#### Apparatus and stimuli

A computer monitor and a keyboard were provided to both sides of a partition and controlled by one main computer. Stimuli and responses were controlled by MATLAB 7.12.0 software (Mathworks, MA, U.S.A). Stimuli were presented on a 21.5-in. LCD monitor (LG Flatron W2261VZ-PF, Korea) with a screen resolution of 1920 × 1080 pixels. Responses were made by pressing the “a” key of a standard keyboard for the white colored car on the left side and the “p” key of another keyboard for the blue colored car on the right side. The cars presented on each side were identical except for the color and direction and were approximately 5 × 3.5 cm in size. A horizontal line with 1.3-cm height was presented right below the cars to give an image of road.

#### Procedure

**Figure 2:**
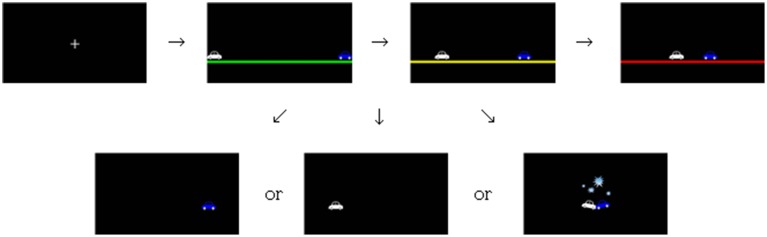
**Procedure of the chicken game experiment**.

The experiment was conducted in a pair of participants who had not met each other before. Participants were not instructed to pursue a certain goal in a game, but were informed that the chicken game tests which person becomes a “chicken.” They were told that they would be paid the summed rewards (costs) of two randomly picked trials from the experimental session in addition to 10,000 KRW. A trial in the game progressed as follows. At the beginning of each trial, a white fixation cross (0.5 × 0.5 cm) was presented for 1 s in the center of the screen with a black background (see Figure 2). Then, two cars with different colors popped up on each horizontal ends of the screen. The car on the left side was assigned to the player seated on the left, and the car on the right side was assigned to the player on the right. A trial was composed of three 1-s intervals. After each interval, the cars approached to each other by 8 cm and eventually crashed after 3 s, unless one or two players avoided before. The color of the horizontal line below the cars was initially green in the first interval, changed to yellow in the second interval, and changed again to red in the third interval, imitating the colors of road signals. The players were to choose to avoid by pressing the key assigned to them at any interval. If any participant chose to avoid at any time during a trial, his/her car on the screen disappeared in the next interval. At the end of every trial, the resulting reward of the trial was shown for 1 s on the screen of each participant. The resulting rewards followed the reward table shown in Figure 1.

After the written and oral instructions about the reward structure and the sequence of the game, 10 practice-trials were conducted. Then, participants were instructed to put in the earplugs so that they could not hear the key-pressing sound of the partner in the main trials. They started the main experimental session by pressing the response keys when they were ready to play. A total of 100 trials of the game were played during the experimental session, with a 1-min break after the first 50 trials.

### Models

Several models of fairness in human behavior associated with fairness were built and compared in the iterated chicken game structure. Most of the previous models had been developed for one-shot games so they were adjusted to iterated games used in the present study. Differences between the models lied in the computation of the utility function of an action. The players shared the same means to calculate the probability of taking an action given the utility functions of a model. As such, here we mainly describe how to compute the utility function of each of three models: Fehr and Schmidt model, Cox's reciprocity model and the proposed self-concept model.

#### Fehr and Schmidt model

We modified the two-player case of the Fehr and Schmidt model to be applicable to an iterated game. In the modified Fehr and Schmidt model, the total reward is the sum of rewards a player has received up to the current trial and the expected reward after the current trial to calculate:

(1) $$\begin{array}{l} E(\Pi_{i})=\sum_{t\;=\;1}^{n\;-\;1}\pi_{i}\left(t\right)+E(\pi_{i}^{a_{i}})\\ E(\Pi_{j})=\sum_{t\;=\;1}^{n\;-\;1}\pi_{j}\left(t\right)+E(\pi_{j}^{a_{i}}) \end{array}$$

*E*(Π_*i*_) is the expected total reward of player *i* after he/she conducts a certain action *a*_*i*_, and *E*(Π_*j*_) is the expected total reward of player *j* after player *i* conducts the action *a*_*i*_. π_*i*_(*t*) is the reward of player *i* at trial *t* and $E(\pi_{i}^{a_{i}})$ is the expected reward of player *i* for the current *n*^*th*^ trial when he/she executes an action *a*_*i*_. As any belief about the other's behavior is not accounted for in this model, $E(\pi_{i}^{A})$ and $E(\pi_{j}^{A})$ are calculated as the average reward following all possible actions *A*. The utility function of action *A* of player *i, u*_*i*_(A) is then given by,

(2) $$u_{i}(\text{A})=\{\begin{array}{l} E(\Pi_{i})-\alpha (E\left(\Pi_{j}\right)-E\left(\Pi_{i}\right)),\text{if }E\left(\Pi_{i}\right)<E(\Pi_{j})\\ E(\Pi_{i})-\beta (E\left(\Pi_{i}\right)-E\left(\Pi_{j}\right)),\text{if }E\left(\Pi_{i}\right)\geq E(\Pi_{j}) \end{array}$$

where β ≤ α and 0 ≤ β < 1. *E*(Π_*j*_)−*E*(Π_*i*_) and *E*(Π_*i*_)−*E*(Π_*j*_) represent a utility loss from an unequal distribution of income. Note that the utility loss from the disparity when a player's own income is lower than that of the opponent, α(*E*(Π_*j*_)−*E*(Π_*i*_)), is larger than or equal to the loss from an advantageous distribution, β(*E*(Π_*i*_)−*E*(Π_*j*_)), because α is no less than β. α and β are free parameters to be fitted to the data (see below for the optimization procedure).

#### Cox et al.'s model

Cox et al.'s (2007) model was modified to be fitted to the present game structure. The emotional state in the original model was simplified by excluding a relative status parameter, and our assumption that the sense of reciprocity is volatile was implemented by a new parameter as follows:

(3) $$f_{i}\left(a_{i}\right)=\frac{\pi_{j}^{h}(a_{i})-E(\pi_{j}^{h})}{E(\pi_{j}^{h}-\pi_{j}^{l})}(\text{normalized})$$

*f*_*i*_(*a*_*i*_) represents player *i*'s kindness to player *j* at each trial. $\pi_{j}^{h}\left(a_{i}\right)$ is the highest payoff possible for player *j* when player *i* chooses an action *a*_*i*_, and $\;E(\pi_{j}^{h})$ is the average of the highest payoff possible for player *j* for every action available for player *i*. A positive value of *f*_*i*_(*a*_*i*_) indicates that player *i* becomes more kind and a negative value indicates that he/she becomes less kind. It is then normalized by $\;E(\pi_{j}^{h}-\pi_{j}^{l})$, the average of the range of player *j*'s payoff given by a difference between the highest and the lowest possible payoff, $\;(\pi_{j}^{h}-\pi_{j}^{l})$ for player *j*. This normalization process ensures *f*_*i*_(*a*_*i*_) to have comparable values when applied to the games with different reward scales. In the chicken game, the value of *f*_*i*_(*a*_*i, j*_) is always 1 if player *i* or *j* avoids and -1 if he/she rushes. That is, one who avoids is always perceived as kind with positive *f*_*i*_ value, while rushing is unconditionally unkind, reflected by the negative value.

Once the kindness value of *f*_*i*_ is calculated, the emotional state of player *i*, θ_*i*_, in the Cox model is computed as follows:

(4) $$\begin{array}{l} r_{i}=f_{j}\left(a_{j}\right)\\ \theta_{i}=\delta r_{i} \end{array}$$

It is assumed that there is no definable difference in the relative status between the participants in the present experiment. So only the reciprocity, not the relative status is considered for the emotional state in Cox et al.'s model. The reciprocity of player *i, r*_*i*_ is equal to the player *j*'s kindness to player *i* given the action of player *j*. δ is the sensitivity to the reciprocity of player *i* that determines the degree to which the emotional state is influenced by the reciprocity (0 ≤ δ ≤ 1). The emotional state of player *i* at trial *t*+1 is then updated as,

(5) $$\theta_{i}\left(t+1\right)=\gamma \theta_{i}\left(t\right)+\beta$$

Based on the present assumption that the sense of reciprocity is volatile, the reciprocity at trial *t* is maintained only to the degree of a retention parameter (γ) with the restriction, 0 ≤ γ ≤ 1. The initial benevolence parameter (−1 ≤ β ≤ 1) is added to the reciprocity updated every trial. An individual with positive β is inclined to show positive reciprocity and help others while a person with negative β tends to show negative reciprocity and harm others.

The utility function of action *a* of player *i, u*_*i*_(*a*_*i*_), at trial *t* is given by,

(6) $$u_{i}\left(a_{i}\right)=\frac{1}{\alpha }\left({\left(\sum_{t\;=\;1}^{n}\pi_{i}\left(t\right)\text{​}+E(\pi_{i}^{a_{i}})\right)}^{\alpha }+\theta_{i}{\left(\sum_{t\;=\;1}^{n}\pi_{j}\left(t\right)+E(\pi_{j}^{a_{i}})\right)}^{\alpha }\right)$$

The modified constant elasticity of the substitution (CES) utility function is used as player i's utility function for his/her action. The convexity parameter α is restricted to 0 < α ≤ 1 because if α is negative, the utility of an action becomes negative even when the income is extremely high. The effect of α varies according to one's emotional state. With a positive emotional state (θ>0), a player prefers an equal distribution of payoffs between the players when α is low, and becomes indifferent to the distribution when α is high. With a negative emotional state (θ < 0), he/she prefers more strongly to have all the payoffs as his/her own with a lower α.

#### Self-concept model

In the self-concept model, players update the degree of reciprocity of themselves as well as that of their opponent's. The basic structure of the model is identical to the modified Cox's model as follows:

(7) $$\begin{array}{l} \;F_{i}^{self}(t+1)=\gamma F_{i}^{self}(t)+f_{i}(a_{i}(t))\\ F_{i}^{other}(t+1)=\gamma F_{i}^{other}(t)+f_{j}(a_{j}(t))+\beta \end{array}$$

(8) $$r_{i}=2(\delta F_{i}^{other}-(1-\delta )F_{i}^{self})$$

Player i's degree of kindness $\;(F_{i}^{self})$ and player j's degree of kindness perceived by player i $\left(F_{i}^{other}\right)$ are updated every trial *t* using the *f*value, where the initial value of F is set to 0. If a player keeps executing an unfavorable action, he/she is perceived as harmful with a negative F value, and a player with helpful behavior is perceived as helpful with a positive F value. γ refers to the rate of retention of reciprocity as above, reflecting the assumption that reciprocity is volatile. The initial benevolence parameter β is added only to the other player's kindness so that an individual with a positive β perceives the other to be more kind, and one with a negative β perceives the other to be less kind. The reciprocity (*r*_*i*_) in this model is defined by a difference between the degree of one's own kindness and that of the other. If a player perceives the other player's degree of kindness as higher than his/her own, positive reciprocity emerges. On the other hand, negative reciprocity happens if a player thinks that he/she was more kind than the other player was. Instead of the sensitivity to the reciprocity parameter in Cox et al.'s model, a relative weight of the other's kindness parameter (0 ≤ δ ≤ 1) is developed to compute the emotion state of player i. If δ is higher than 0.5, a person cares more about other people's kindness toward him/her than his/her kindness to others. In Equation (8), the resulting difference between weighted F values is doubled such that *r*_*i*_ when the relative weight is equal between self and the other (i.e., when δ is 0.5) to become the reference $\left(F_{i}^{other}-F_{i}^{self}\right)$ which maintains initial F values from Equation (7). The utility function of an action is identical to that of the modified Cox's model:

(9) $$u_{i}\left(a_{i}\right)=\frac{1}{\alpha }\left({\left(\sum_{t\;=\;1}^{n}\pi_{i}(t)+E(\pi_{i}^{a_{i}})\right)}^{\alpha }+r_{i}{\left(\sum_{t\;=\;1}^{n}\pi_{j}(t)+E(\pi_{j}^{a_{i}})\right)}^{\alpha }\right)$$

#### Probability of an action

For all the models introduced above, the utility of all possible actions was compared and an action with the highest expected utility was chosen. The probability of an action for the next trial was then calculated as follows:

(10) $$\begin{array}{l} p_{i}(avoid_{i}\;(t+1))=sigmoid(u_{i}\;(\text{avoid})-u_{i}\;(\text{rush}))\;\\ \;sigmoid\;(z)=\frac{1}{1+e^{-z}} \end{array}$$

*p*_*i*_(*avoid*_*i*_ (*t* + 1)) is a probability that player i chooses avoidance as his/her next action. It increases with a higher utility of avoidance and decreases with a lower utility. The probability of choosing an action is calculated by the logistic sigmoid function, *sigmoid*(*z*).

### Model evaluation

#### Model fitting

The probabilities of action predicted by the models were fitted to the behavioral data to the way it maximizes the logistic log likelihood (log L) of the prediction.

(11) $$\log L=\sum_{t\;=\;1}^{n(trial)}\sum_{i\;=\;1}^{n(player)}\log\;p\left(a_{i}(t)\right)$$

The fmincon function in the optimization toolbox of MATLAB 7.12.0 was used to define the parameters that maximize the logistic log likelihood. The models were then evaluated by Bayesian Integration Criterion (BIC) score with fitted parameters,

(12) BIC=−2logL+k · log(n(trial))

where k indicates the number of parameters of a model. A model with many parameters tends to show high likelihood, but an increasing number of parameters may result in overfitting. Because the BIC score penalizes free parameters, it is appropriate to use this method for the comparison of the models that have different numbers of parameters. A low BIC score refers to high likelihood of a model. In the present analyses, the BIC scores of the models were averaged over the pairs of participants.

#### Non-stationary parameter analysis

To observe parametric changes through the process of the games, the optimal parameters that maximize the logistic log likelihood in a smaller trial-window was calculated. The window size was arbitrarily determined as 20 trials. The parameters were fitted to the first to 20th trial, then to the second to 21st trial, and then to n to n + 19 trial by the end of the trials. A specific method used to extract the parameters of each model was same as before. With the fitted parameters, the difference of the parameters between the two players of each pair was calculated for every 20-trial window along the game. Then, it was tested if the changes in these parameter differences over time were correlated with the BIC scores that were also calculated in each window with the fitted parameters. Because the models showed lower BIC scores and a better prediction when they predicted distinct behavioral patterns, it was assumed that if the differences of parameters and BIC scores were correlated, the changes in parameters reflected the evolution of consensus that led to distinct behavioral patterns.

## Results

### Behavioral data

**Figure 3:**
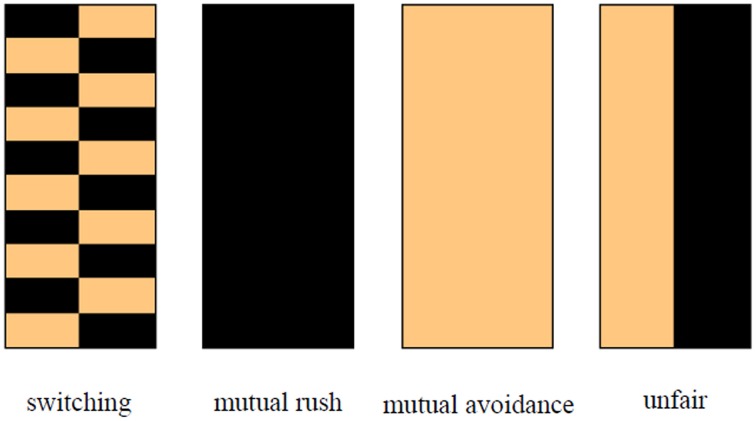
**Illustration of the four simple symmetric behavioral patterns**. Light colored block indicates an avoidance trial, and a dark colored one indicates a rush trial. The left and the right columns represent the behavior of Players 1 and 2, respectively.

In a two-trial window, there are four simple symmetric patterns of behavior that can appear in the chicken game. One is a “switching” pattern in which one player rushes and the other player avoids in a trial and switches their role in the next trial. A “mutual rush” pattern and a “mutual avoidance” pattern consist of a pair of players who consistently rush or avoid. In an “unfair” pattern, one player avoids and the other player rushes repeatedly. These patterns are illustrated in Figure 3. The left half of each picture shows player 1's action and the other half shows that of player 2. The light and dark colors indicate an avoidance and a rush trial, respectively.

The numbers of these two-trial window patterns in the behavioral data were counted to categorize the behaviors. The behavioral data of a pair of participants was defined to have a distinct pattern if the number of the trials showing a same specific pattern exceeded a threshold. Thresholds were determined by the simulation in the self-concept model with uniformly distributed random parameters. The 50,000th largest number of each pattern obtained from the 1,000,000 simulations was defined as a threshold: as a result, it was 24 out of 100 trials for the “switching” pattern, 22 for the “mutual avoidance” pattern, 23 for the “mutual rush” pattern, and 20 for the “unfair” pattern, respectively. The number of the “unfair” pattern produced by extreme values of parameters was relatively lower than that of the other patterns.

In the behavioral data, the most frequently observed pattern was a “switching” pattern. A fairly clear and consistent switching pattern was found for 12 out of 36 pairs of participants. In most cases, the switching pattern sustained steadily once it was generated. The “mutual rush” pattern was dominant for nine pairs of participants who repeatedly crashed their cars. On the contrary, two pairs of participants with the “mutual avoidance” pattern consistently avoided each other. The “mutual rush” pattern was likely to be maintained through the whole game while the “mutual avoidance” pattern was hardly sustained for a long period. The least frequent but still clear pattern was the “unfair” pattern. In this pattern, one player kept rushing the car while the other player continuously avoided. The pattern was found for only one pair of players. The “unfair” pattern was dominant through the whole game of that pair. The other 12 pairs did not display any consistent pattern based on the thresholding scheme. Players in those pairs showed seemingly random behaviors hardly explained by a model.

### Model fitting and simulations

**Figure 4:**
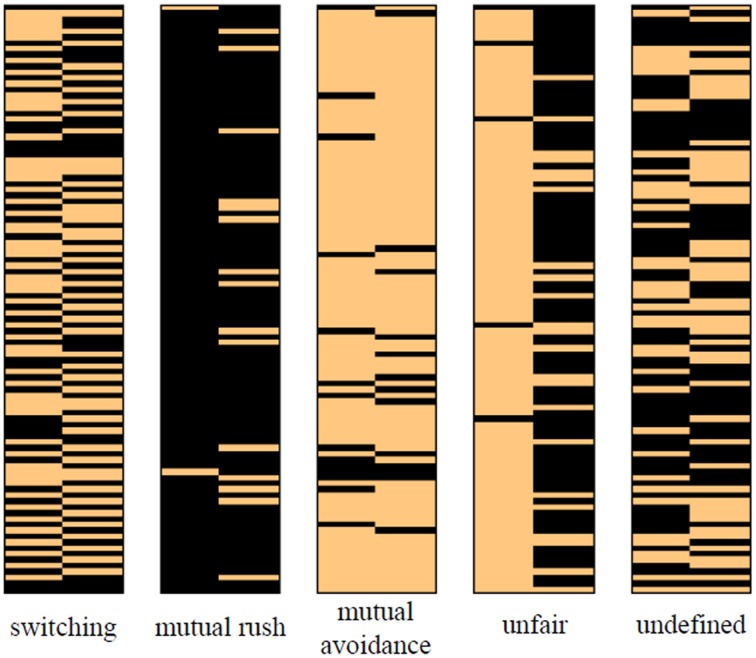
**Simulated behavioral patterns using the self-concept model with the average parameters of the players in each pattern**.

The parameters in the models were fitted to the data in a way that maximizes the likelihood (See Model Fitting). Because the parameter values of a player imply a certain strategy in a game that leads to different behavioral patterns with a different opponent's strategy, the parameters of Players 1 and 2 in the pairs with the same behavioral pattern were averaged, respectively. The fitted values of the models are shown in Table 1. In the simulations using the parameters in Table 1, the self-concept model successfully generated the patterns. The number of two-trial windows with distinct patterns was counted for each pattern over 100 trials of the simulation. On average from 10,000 simulations, there were 43.12 (standard deviation (*SD* = 7.13) switching patterns, 66.11 (*SD* = 7.27) mutual avoidance patterns, 46.29 (*SD* = 10.85) mutual rush patterns, and 55.28 (*SD* = 6.27) unfair patterns out of 99 two-trial windows in the 100 trial simulations, respectively, when the parameter values for a certain behavioral pattern were used. A sample of simulated behavioral pattern is shown in Figure 4.

**Table 1:** **Average model parameters of the players with certain behavioral patterns**.

**(A) Fehr-Schmidt model**.								
	**Player 1**				**Player 2**			
		**alpha**		**beta**		**alpha**		**beta**
Switching		0.2301		0.3642		0.0863		0.1695
Rush		0.0538		0.5824		0.0013		0.2924
Avoid		0.0586		0.2500		0.0475		0.2500
Unfair		0.0187		0.0000		0.0187		0.0117
Undefined		0.0719		0.261		0.0483		0.1168
**(B) Cox et al.'s model**								
	**Player 1**				**Player 2**			
	**alpha**	**beta**	**gamma**	**delta**	**alpha**	**beta**	**gamma**	**delta**
Switching	0.9904	−0.0653	0.0264	0.9872	0.9911	−0.0213	0.0357	0.9787
Rush	0.9031	−0.7186	0.5791	0.6693	0.7820	−0.5580	0.4555	0.7516
Avoid	0.9893	0.1341	0.3645	0.9945	0.9999	0.2317	0.3545	0.9996
Unfair	0.9435	0.9130	0.7067	0.0301	0.6106	−0.4942	0.932	0.1556
Undefined	0.9123	−0.1407	0.3300	0.8087	0.8635	−0.0684	0.4038	0.7397
**(C) Self-concept model**								
	**Player 1**				**Player 2**			
	**alpha**	**beta**	**gamma**	**delta**	**alpha**	**beta**	**gamma**	**delta**
Switching	0.9764	−0.0689	0.1102	0.6099	0.9650	−0.0657	0.0638	0.6494
Rush	0.8632	−0.7388	0.6731	0.6965	0.7860	−0.4299	0.4423	0.7893
Avoid	0.8648	0.1266	0.3703	0.9786	0.9968	0.1704	0.2729	0.9171
Unfair	0.6443	0.9683	0.9303	0.9316	0.9112	−0.8921	0.1993	0.9905
Undefined	0.8256	−0.2006	0.5741	0.6464	0.7488	−0.0681	0.4533	0.7824

### Model evaluation

**Figure 5:**
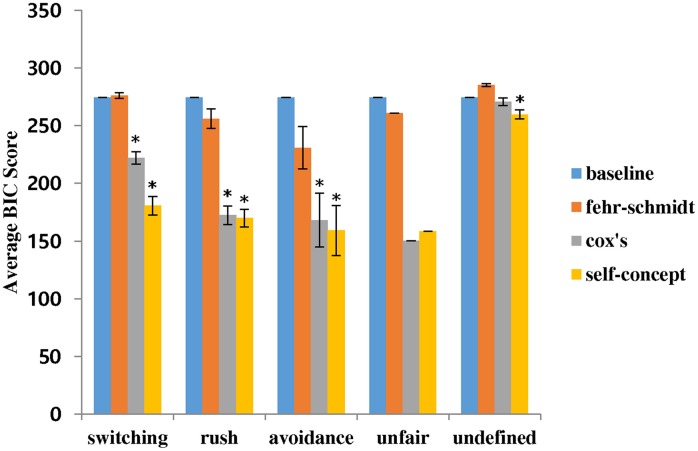
**BIC scores of the models for each behavioral pattern**. Error bars indicate standard errors. Asterisks refer to the significant difference from the baseline at 5% level.

Figure 5 shows the BIC scores of the models for each group of pairs with the distinct patterns averaged by the number of pairs. Those averaged BIC scores were compared by the paired *t*-test. The baseline was defined as the BIC score when the probabilities of actions for every trial were predicted as 50% each, to test if the models' BIC scores are lower than the score at the chance level prediction. The summary of comparison with the baseline is shown in Table 2. The Fehr and Schmidt model showed a low explanatory power for overall behavioral patterns from every pair of participants. Overall BIC score of this model (*M* = 271.66, *SD* = 32.17) was not significantly different from the baseline prediction (*M* = 277.26, *SD* = 0), *p* = 0.6012. Cox et al.'s model and the self-concept model both decently predicted all the distinct patterns. They explained better than the Fehr and Schmidt model and the baseline prediction for every distinct pattern (*p*s < 0.01). The overall BIC score of the self-concept model (*M* = 204.11, *SD* = 60.37) was significantly lower than that of Cox et al.'s model (*M* = 222.42, *SD* = 55.68), *p* < 0.01. Specifically, the self-concept model explained the switching pattern and undefined pattern significantly better than Cox et al.'s model (*ps* < 0.01). The self-concept model also showed a lower BIC score at the mutual rush and avoidance patterns than Cox et al.'s model but the difference was not statistically significant (*p*s = 0.1347 and 0.2275, respectively). Note, however, that the *t*-test on the BIC scores of the avoidance pattern was not reliable because there were only two samples. Similarly, the BIC scores of the unfair pattern were inappropriate for the *t*-test as there was only one pair with an unfair pattern. In addition, the self-concept model was the only model with a BIC score for undefined patterns significantly lower than that of the baseline prediction, *p* = 0.0434. The Fehr and Schmidt model also showed significant difference in undefined patterns, *p* = 0.0125, but the BIC score was higher than the baseline, implying that the model predicts worse than at chance level. No other significant difference was found between the models.

**Table 2:** **The results of the *t*-test between the BIC scores of the models and the baseline (BIC = 277.26)**.

	**Overall**		**Switching**		**Rush**		**Avoid**		**Unfair**		**Undefined**	
	**BIC**	***t(p)***	**BIC**	***t(p)***	**BIC**	***t(p)***	**BIC**	***t(p)***	**BIC**	***t(p)***	**BIC**	***t(p)***
Fehr-Schmidt	271.66 (32.17)	−1.04 (0.83)	276.15 (17.98)	−0.21 (0.83)	256.03 (50.75)	−1.26 (0.24)	230.81 (51.83)	−1.27 (0.43)	260.68 (−)	−	285.14 (9.69)	2.93 (0.01)^*^
Cox	222.42 (55.68)	−5.91 (0.00)^**^	222.16 (37.53)	−5.10 (0.00)^**^	172.35 (47.33)	−6.65 (0.00)^**^	168.11 (66.11)	−2.34 (0.26)	150.42 (−)	−	270.64 (23.91)	−1.00 (0.34)
Self-concept	204.11 (60.37)	−7.27 (0.00)^**^	180.61 (55.05)	−6.10 (0.00)^**^	169.89 (46.82)	−6.88 (0.00)^**^	159.19 (61.39)	−2.72 (0.22)	158.60 (−)	−	259.79 (27.90)	−2.26 (0.04)^*^

** *significant at 1% level*.

* *significant at 5% level*.

*The number in the brackets in the BIC score column is the standard deviation*.

### Non-stationary parameter analysis

**Figure 6:**
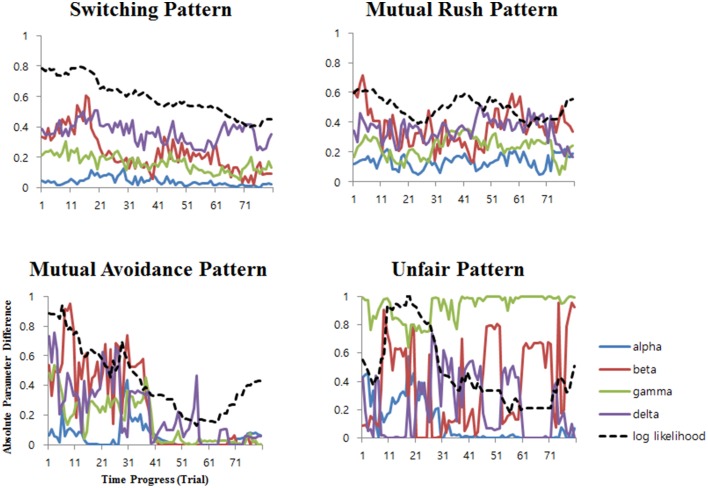
**Differences of the parameters between two players in the self-concept model as a function of time progress**. The dashed line represents the additive inverse of the log likelihood value (-logL), which is proportional to the BIC score (see Equation 12).

For Cox et al.'s model and the self-concept model, which appeared to explain the behavior patterns better than the baseline, the non-stationary parameter analysis was conducted. Among the 81 sets of 20-trial time windows fitted with the models, *t*-test analysis revealed significant changes in the model parameter difference between the players from the first 40 to the second 40 of the time windows (*ps* < 0.01) in all the distinct patterns except for the mutual rush pattern, as shown in Table 3. In particular, the players' difference of parameters significantly decreased for every parameter in the switching and the avoidance patterns. The temporal patterns of averaged parameter differences in the self-concept model and its corresponding BIC scores are depicted as a function of time progress in Figure 6. The difference of the parameters between the players in the self-concept model showed a strong correlation with BIC scores for most of the parameters (see Table 4). Mostly, the BIC scores decreased with decreasing difference in the parameters (positive correlation). However, the initial benevolence parameter (β) in the mutual rush and unfair patterns had no significant correlation with the BIC scores. Although the results of the non-stationary parameter analysis for Cox et al.'s model was similar to that of the self-concept model, there were seven parameters with no significant correlation with BIC score in Cox et al.'s model, while there were five in the self-concept model.

**Table 3:** **The results of the *t*-test between the players' difference of parameters in the first half and the second half of the trial windows**.

	**alpha**		**beta**		**gamma**		**Delta**	
	***t***	***p***	***t***	***p***	***t***	***p***	***t***	***p***
**(A) Cox et al.'s model**.								
Switching	−12.52^**^	0.00	−3.78^**^	0.00	−13.38^**^	0.00	−12.78^**^	0.00
Rush	2.55^*^	0.02	0.72	0.47	0.81	0.42	−1.22	0.23
Avoid	−7.90^**^	0.00	−14.26^**^	0.00	−9.91^**^	0.00	−11.38^**^	0.00
Unfair	−1.95	0.06	3.91^**^	0.00	4.55^**^	0.00	−7.55^**^	0.00
**(B) Self-concept model**								
Switching	−6.09^**^	0.00	−7.22^**^	0.00	−7.75^**^	0.00	−4.31^**^	0.00
Rush	1.51	0.14	0.41	0.69	0.81	0.42	1.56	0.13
Avoid	−4.62^**^	0.00	−15.15^**^	0.00	−14.14^**^	0.00	−9.19^**^	0.00
Unfair	−7.89^**^	0.00	3.06^**^	0.00	7.12^**^	0.00	−1.01	0.32

** *significant at 1% level*.

* *significant at 5% level*.

**Table 4:** **Correlation between the difference of parameters and the log likelihood of each behavioral pattern**.

**(A) Cox et al.'s model**								
	**alpha**		**Beta**		**Gamma**		**delta**	
	***r***	***p***	***r***	***p***	***r***	***p***	***r***	***p***
Switching	0.7956^**^	0.0000	0.8704^**^	0.0000	0.4423^**^	0.0000	0.8394^**^	0.0000
Rush	0.1629	0.1489	0.3095^**^	0.0052	0.2525	0.0238	−0.0308	0.7867
Avoid	0.5670^**^	0.0000	0.7478^**^	0.0000	0.7961^**^	0.0000	0.5600^**^	0.0000
Unfair	0.0466	0.6817	−0.3325^**^	0.0026	−0.3466^**^	0.0016	0.6591^**^	0.0000
Undefined	−0.1037	0.3611	−0.0171	0.8799	−0.4869^**^	0.0000	−0.2060	0.0668
**(B) Self-concept model**								
Switching	0.4767^**^	0.0000	0.7156^**^	0.0000	0.8056^**^	0.0000	0.4209^**^	0.0001
Rush	0.2608^*^	0.0195	0.3806^**^	0.0005	0.1721	0.1270	−0.0286	0.8014
Avoid	0.4012^**^	0.0002	0.7498^**^	0.0000	0.7704^**^	0.0000	0.6410^**^	0.0000
Unfair	0.7963^**^	0.0000	−0.6972^**^	0.0000	−0.0227	0.8420	−0.0993	0.3810
Undefined	0.3541^*^	0.0013	−0.4797^**^	0.0000	0.3886^**^	0.0004	0.1302	0.2500

** *significant at 1% level*.

* *significant at 5% level*.

## Discussion

Extant models did not usually consider the role of one's own kindness when regarding reciprocity. Because many economic games used in experiments have a bilateral structure, we proposed a new model with a variable catching an emotion aroused by the kindness of oneself to the other in addition to the other's kindness to oneself. In the chicken game used in the present study with all the models modified to be applicable to an iterated game, various behavioral patterns were best explained by the newly proposed self-concept model.

### Behavioral pattern explanation

Four dominant patterns of behavior between two players were found from the present behavioral data. It is notable that the behavior in the most frequent “switching” pattern is unfair in a one-shot game because only one player has a positive payoff and the other player suffers from a negative payoff. However, the resulting payoff of the iterated switching pattern reaches to the equal distribution. This is a pattern that can be found only in iterated games, and it reflects the reciprocal behavior of the players. A player who avoided in a trial would be predisposed to rush for the next trial as she/he had negative reciprocity, whereas an individual who rushed in a trial would be inclined to avoid in the next trial because she/he had positive reciprocity.

The self-concept model showed an especially better prediction for the switching pattern, compared to the other models. A possible explanation is that a player's negative reciprocity is strengthened by positive self-concept if he/she avoids when his/her counterpart rushes, and the positive reciprocity is also strengthened by negative self-concept if he/she rushes when the other player avoids. One of the main differences between Cox et al.'s model and the self-concept model occurs at this point. In Cox et al.'s model, the negative (positive) reciprocity from an unfair trial that a player avoids (rushes) while the other player rushes (avoids) is equal to the negative (positive) reciprocity aroused by mutual rush (avoidance). However, it is natural to have a more negative feeling when a player suffers from one-sided unkindness than from mutual hostility, because of inequality in outcome distribution (Tricomi et al., 2010).

Unlike the switching pattern, the mutual rush, and mutual avoidance patterns are explained by one-shot fairness. Although the mutual rush and the mutual avoidance patterns both have the equal distribution of payoff, the mutual avoidance pattern provides a higher payoff to the players than the mutual rush pattern does. However, only two pairs showed the mutual avoidance pattern while nine pairs revealed the mutual rush pattern. It is worth noting that the resulting payoffs are the same between the mutual avoidance pattern and the switching pattern. People may just prefer the switching pattern to the avoidance pattern if they are getting the same payoff. If it were not for the switching pattern that occupied 12 pairs of participants out of 36 pairs, there might have been much more mutual avoidance pattern, even more than the mutual rush pattern. It is reasonable to choose the switching pattern instead of the mutual avoidance pattern to guarantee a stable pattern. Betrayal is highly desirable in the mutual avoidance pattern because a player who betrays and rushes gets more reward. However, in the switching pattern, a player who rushes when it is his/her turn to avoid also loses his/her own payoff. Therefore, establishing the switching pattern is advantageous to prevent betrayal. This is consistent with recent studies suggesting that people are averse to betrayal besides the monetary loss (Bohnet and Zeckhauser, 2004; Aimone and Houser, 2011, 2012).

The “unfair” pattern is incompatible with altruism because it propagates the unequal distribution of payoff further and further. The pattern is inconsistent with the prediction based on reciprocity, too. The players in this pattern do not help the other who is being kind to them nor punish the other with selfish behavior. However, the pattern is explained by initial benevolence in the present model. If a person with extremely high initial benevolence meets the other with extremely low initial benevolence, the unfair pattern could occur (see Table 1). Because of extreme initial benevolence, they are strongly inclined to do a specific pattern of behavior regardless of other people's interest or kindness (e.g., Rushton, 1984). The unfair pattern refutes the assumption that people care about other people, but only one pair showed this pattern in the present study.

In addition, the behaviors of 12 pairs of the participants that did not show a repeating pattern were classified as the “undefined” pattern. Only the self-concept model succeeded to explain those patterns better than the baseline prediction. There may still be logic inside the seemingly random patterns, and it is likely that the unilateral reciprocity is not enough to explain those patterns. The self-concept seems to play a certain role in an undefined pattern.

Although all types of response patterns are explained by the present model, the contribution of the self-concept in decision-making is not always high. For example, Player 1's average delta (δ) value 0.6099 in the switching pattern shown in Table 1 indicates that the player relied on reciprocity by about 61% and the self-concept influenced the rest of 39% in the decision of the actions. In line with the other models of fairness (e.g., Rabin, 1993; Cox et al., 2007), the main determinant of action was reciprocity, which is partially modulated by the self-concept. Furthermore, the players in the mutual avoidance and unfair patterns showed delta (δ) values higher than 0.9 on average, indicating that some players were not sensitive to their self-image and concentrated much more on the opponent's behavior. According to Mazar et al. (2008), who addressed that the self-concept is not updated when people are inattentive to their own moral standard of themselves, some players who are ignorant of the fairness of their behavior would not adjust their self-concept based on their action in a game. Especially, Player 2 in the unfair pattern showed an extremely high delta (δ) value, implying that he/she denied negative self-image from his/her unfair action of continuously rushing against the opponent who avoided. The characteristic that inclines the player to selfish behavior is also reflected by a highly negative “initial benevolence” parameter (β) value. Also, players in the mutual avoidance pattern maintained mutual kindness by minimizing the effect of positive self-concept that arouses moral-licensing that may result in betrayal. That is, certain players continuously ignored their self-concept to maintain their behavioral pattern, either it is kind, or unkind.

However, there were only three pairs of the players who were insensitive to self-concept, resulting in the mutual avoidance or unfair pattern. The result that the majority of the players in the other patterns had a relatively low delta (δ) value implies that people usually consider the self-concept when deciding their next behavior. The players tended to become unkind after a kind behavior, and vice versa. This finding supports the theory that people adjust their behavior to maintain their self-concept within an ideal level determined by personal moral standard (Sachdeva et al., 2009). In the present model, the standard level of morality, or fairness is the opponent's kindness, because the players' self-concept is modulated when they feel that they are relatively more kind or unkind than the opponent. This is consistent with a widespread assertion that the self-concept and the moral standard in social interaction is not completely internal (Shrauger and Schoeneman, 1979). That is, one's own self-concept reflects the imagination about other people judge oneself (e.g., Schneider, 1970; Raven and Rubin, 1976).

### Process of non-stationary parameter

Every player in a game has their own initial rule that decides their behavior, but it is not always in accordance with the other player's rule (Stasser and Titus, 1985). There must be a process of negotiating between the players before they reach a consensus to share a rule. Different initial rules of the players converge to a common rule if they succeed to achieve consensus after experiencing some trials (Stasser, 1988). The non-stationary parameter analyses were conducted to see a process of rule convergence as the game continues. The parameters in the self-concept model reflect a specific rule of a player. For example, a low convexity reflects a rule to be more concerned about the equal distribution of payoff, high initial benevolence implies a rule to be kind regardless of reciprocity, and a low retention rate leads to a rule to forget the past quickly and focus on the emotion from the most recent behaviors. If the players in a game gradually reach a consensus to share a rule, the parameters also change gradually according to a forming rule.

The result of the non-stationary parameter analysis was consistent with the assumption that the shared rules are formed as time goes on. For many patterns, the parameters of the players became similar to each other as the game proceeded so that the difference of parameters gradually converged to zero as shown in Table 3 and Figure 6. In addition, the explanatory power of the model showed a significant correlation with the difference of the parameters (see Table 4). As the parameters of the players became similar, distinct patterns occurred more often and the BIC scores decreased. That is, the model predicted the behavior better when the players' parameters were shared between them. One exception is the unfair pattern, where the parameter values of “beta” are extremely different among two players. This is because the pattern arises mostly from oppositely extreme initial benevolence parameter (β) that leads the players to the contrary behaviors. Also, large difference of retention parameter (γ) in “unfair” pattern in Tables 1B,C indicates that one player with low β deviates from previous behaviors to pursue selfish interest. These differences are also reflected in non-stationary analysis where the differences of β and γ) among two players are enlarged as time goes on (see Table 3).

As shown in Table 4, all the parameters in the switching pattern and the mutual avoidance pattern showed a strong correlation with the explanatory power. The difference between the parameters showed a linear trend of decreasing as the game proceeded and so did the BIC score. It is well fitted to the present assumption that the two players share a rule gradually as the game continues. The switching pattern and the mutual avoidance pattern reflect the process of the rule establishment resulting from a consensus between the players. The parameters of the players of those patterns converged to a certain point as the game proceeded, implying that they reached a consensus to have the same rule. In addition, consistent with the previous suggestion that betrayal is highly desirable in the mutual avoidance pattern, the BIC score of the mutual avoidance pattern started to increase from the last 20 trials of the game. This result indicates a broken consensus when the players try to betray the others to get more reward of their own, as usually seen in Prisoner's Dilemma (e.g., Komorita and Mechling, 1967; Kershenbaum and Komorita, 1970).

The patterns of parameters and the explanatory power were not stable in the mutual rush pattern and the unfair pattern. The differences between the players in the retention parameter (γ) and the sensitivity to the other (δ) parameter showed no significant correlation with the BIC score in those patterns. Further, the changes of the explanatory power were not linear as those of the switching pattern were. Overall, the process of the mutual rush pattern and the unfair pattern was unstable compared to that of the switching pattern and the mutual avoidance pattern.

A shared rule that generates the switching and mutual avoidance patterns is regarded as a good consensus because it maximizes the joint income of the players and the distribution is equal. It is a desirable state for the people who act based on the utility function provided by the self-concept model. On the other hand, the mutual rush, and unfair patterns are disadvantageous because the payoff is very low in the mutual rush pattern and the distribution is extremely unequal in the unfair pattern. It is likely that people continuously try to go against the rule that makes the mutual rush pattern and the unfair pattern, even after a shared rule is formed (e.g., Harrison and McCabe, 1996). This is a possible reason why the rules were not stably established and maintained in the mutual rush and the unfair patterns.

At first, it was assumed that the players who failed to share a rule generate undefined patterns. Thus, it is surprising that the parameters in the undefined patterns also showed a significant correlation with the BIC score (see Table 4). Further, the BIC score of the undefined patterns tended to decrease slightly as the game proceeded. It implies that a seemingly undefined pattern also has some process of negotiation between the players that slowly leads to a stable pattern. If they have more time to establish a common rule, they might have reached a consensus that makes a distinct pattern.

It has been suggested that non-verbal signals express the intention of the signaler, and the behaviors are determined in interactions with the recipient (Dawkins and Krebs, 1978; Caryl, 1979). That is, the behaviors are modulated by the expectation about the other people's behavior according to the interpretation of non-verbal signal (Hinde, 1985). In the present study, the players often tried to influence each other by expressing their internal state through their behavior. For example, in the mutual rush pattern some players started to avoid at some trials, presumably asking for reconciliation. The motivational states conveyed by a signal are then assessed by the behaviors that follow (e.g., Tinbergen, 1959; Baerends, 1976). The receiver of the signal may respond by avoiding, or ignoring it by keeping rushing after the interpretation. The players continuously send, receive, and respond to the signal from the behavior and decide their next action. The non-stationary parameter analyses demonstrate the process of negotiation through the signal. The finding that the behaviors gradually became more predictable suggests that people usually establish a common rule to understand each other through the interactions (e.g., Penrod and Hastie, 1980; Stasser and Davis, 1981).

## Conclusions

The results showed that the phenomena in the chicken game are well explained by the model in which one's own kindness is considered. The concept of one's own kindness is applicable to other types of games. For example, in Harbaugh and Krause (2000) study, the result of an iterated public good game experiment revealed a general pattern that the players' contribution to public good increased at first, but started to decrease at a certain point. Furthermore, participants who made a large contribution in the last iteration of the public good experiment did not share their reward much in a dictator game that was conducted right after the last trial of the public good game. The authors attributed the result to a confusion of the participants who did not clearly understand the reward structure of the games. However, it is possible that the results reflect the concept of one's own kindness (Bodner and Prelec, 2002). If the players made a large enough contribution in the previous game, they would feel morally licensed to make a lower contribution afterwards.

The self-concept determined by one's own kindness is especially important for a game such as the dictator game in which reciprocity plays a small role because the receiver does not have any chance to help or punish the dictator (Cason and Mui, 1998). There must be a fluctuating pattern in the players' behavior in an iterated dictator game if the balancing of self-concept indeed influences decision-making. For example, a player who took most of the payoff in a trial may provide a generous offer next time to offset the negative self-concept from the previous behavior. It would be meaningful to find an implication in the variations of the offer in the game that may have not been interpreted.

The comparison of the models showed that a simple inequality averseness of payoff was not enough to explain the dynamic patterns observed in the chicken game. The model that incorporates the reciprocity and the self-concept provided a much better prediction of the behaviors. The result indicates that people care about their own kindness to others in addition to other people's kindness toward them (e.g., Benabou and Tirole, 2003). However, the main determinant of the behavior was reciprocity in the present model, although it was modulated by the self-concept to some degree. Therefore, the self-concept model complements various models of fairness that include the concept of reciprocity, rather than contradicting them. The findings in the non-stationary parameter analyses were consistent with the assumption that the players in a game gradually establish a common rule that determines the way they act. It implies that people influence each other with non-verbal communication that signals their internal state during the game and reach a consensus at some point. The pattern was more stable when the rule was mutually beneficial than when it was unfair or mutually harmful, suggesting that people always have a motivation to change a rule when the current one is not satisfactory. The present model has the potential to be applied to other games that are seemingly affected by the self-concept, but have not been interpreted in that way. It would be especially effective in games where the effect of reciprocity is minimized and the players mostly concentrate on their own behavior.

## Funding

This work was supported by the National Research Foundation of Korea Grant funded by the Korean Government (NRF-2012R1A2A2A04047239).

### Conflict of interest statement

The authors declare that the research was conducted in the absence of any commercial or financial relationships that could be construed as a potential conflict of interest.
